# COVID-19 in Infants and Children under 2 Years—Could Lung Ultrasound Score Be Correlated with Biomarkers and Symptoms?

**DOI:** 10.3390/biomedicines11102620

**Published:** 2023-09-24

**Authors:** Emil Robert Stoicescu, Jovan Lovrenski, Roxana Iacob, Simona Cerbu, Daniela Iacob, Emil Radu Iacob, Septimiu Radu Susa, Ioana Mihaiela Ciuca, Laura Andreea Bolintineanu (Ghenciu), Andreea Ciornei-Hoffman, Cristian Oancea, Diana Luminita Manolescu

**Affiliations:** 1 Department of Radiology and Medical Imaging, ‘Victor Babes’ University of Medicine and Pharmacy Timisoara, Eftimie Murgu Square No. 2, 300041 Timisoara, Romania; stoicescu.emil@umft.ro (E.R.S.); dmanolescu@umft.ro (D.L.M.); 2 Research Center for Pharmaco-Toxicological Evaluations, ‘Victor Babes’ University of Medicine and Pharmacy Timisoara, Eftimie Murgu Square No. 2, 300041 Timisoara, Romania; 3 IOSUD/Ph.D. School, ‘Victor Babes’ University of Medicine and Pharmacy Timisoara, Eftimie Murgu Square No. 2, 300041 Timisoara, Romania; 4 Faculty of Medicine, University of Novi Sad, Hajduk Veljkova 3, 21000 Novi Sad, Serbia; 5 Institute for Children and Adolescent Health Care of Vojvodina, Hajduk Veljkova 10, 21000 Novi Sad, Serbia; 6 Department of Neonatology, ‘Victor Babes’ University of Medicine and Pharmacy Timisoara, Eftimie Murgu Square No. 2, 300041 Timisoara, Romania; 7 Department of Pediatric Surgery, ‘Victor Babes’ University of Medicine and Pharmacy, Eftimie Murgu Square 2, 300041 Timisoara, Romania; 8 Pediatric Department, ‘Victor Babes’ University of Medicine and Pharmacy Timisoara, Eftimie Murgu Square No. 2, 300041 Timisoara, Romania; 9 Department of Functional Sciences, ‘Victor Babes’ University of Medicine and Pharmacy Timisoara, Eftimie Murgu Square No. 2, 300041 Timisoara, Romania; 10 Department of Anatomy and Embryology, Morphological Sciences, Iuliu Hatieganu University of Medicine and Pharmacy, 400349 Cluj-Napoca, Romania; 11 Department of Radiology and Medical Imaging, County Clinical Emergency Hospital, 400347 Cluj-Napoca, Romania; 12 Center for Research and Innovation in Precision Medicine of Respiratory Diseases (CRIPMRD), ‘Victor Babeș’ University of Medicine and Pharmacy, 300041 Timișoara, Romania; 13 Department of Pulmonology, ‘Victor Babes’ University of Medicine and Pharmacy, 300041 Timisoara, Romania

**Keywords:** lung ultrasound, infants, children, COVID-19, SARS-CoV-2, multisystem inflammatory syndrome

## Abstract

Introduction: It is already well known that infants and children infected with COVID-19 develop mild to moderate forms of the disease, with fever and oropharyngeal congestion being the most common symptoms. However, there are instances when patients claim to be experiencing respiratory symptoms. Because of the repeated lung examinations required in these situations, non-irradiating imaging techniques are preferred. This study’s objective is to ascertain the value of lung ultrasonography (LUS) in the medical management of these specific cases. Methods: Infants and children under two years old with SARS-CoV-2 infection were evaluated using LUS. Patients with other respiratory pathologies were excluded by using specific tests. The LUS score (LUSS) was correlated with biomarkers and clinical findings using the Mann–Whitney U test and Spearman’s rank correlation rho. Results: The LUSS for each patient varied from 1 to 8 points out of a maximum of 36 points. The arithmetic mean was 4.47 ± 2.36 (S.D), while the 95% CI for the arithmetic mean was 3.33 to 5.61. Sparse B-lines were present in all enrolled infants and children (100%), while only 36.84% developed alveolar syndrome (confluent B-lines). The lung changes were correlated with their biomarkers, specifically inflammatory markers. The correlation between LUSS and LDH, D-dimers, and IL-6 was a strongly positive one with rho = 0.55 (*p =* 0.001, 95% CI 0.13 to 0.80) between the LUSS and D-dimer levels and *rho* = 0.60 (*p =* 0.03, 95% CI 0.04 to 0.87) between LUSS and D-dimer levels at symptomatic infants and children (with respiratory involvement). Conclusions: Infants and children under the age of two are prone to develop mild forms of COVID-19 disease with a B-line pattern on LUS, although inflammatory markers have elevated blood levels. Despite the small sample, D-dimer levels and O_2_ saturation were correlated with LUSS in patients with respiratory involvement, while similar results were also found in the entire lot.

## 1. Introduction

Nowadays, with the advancement in technology, the thoracic ultrasound technique is considered to be a useful tool in the exploration of respiratory pathologies, especially in children, infants and newborns, following the ALARA principles (as low as reasonably achievable) [1,2,3]. Given the advantages of being a non-irradiating and non-invasive method, this technique could replace conventional radiography in the detection of subpleural injuries of the lung [2,4]. Ultrasonography is accessible and repeatable, and it can also be used at the patient’s bedside for diagnosis, prognosis, and monitoring of infants with lung injuries [5,6]. Lovrenski concludes that the most important feature of lung ultrasound (LUS) is that it does not lie—if something can be found by this imaging technique, it is there [2].

The SARS-CoV-2 infection seems to be suitable for this kind of examination because the commonly affected areas are the subpleural and posterior lung fields, which can be optimally explored by using this technique [7]. Campagnano et al. conclude that lung ultrasound has higher sensitivity than computer tomography in detecting lesions in the inferior and posterior fields of the lung in subpleural regions where COVID-19-associated pneumonia is often expressed as alveolar-interstitial injury [7]. Additionally, high-resolution computer tomography (HRCT) has proven to be more effective in evaluating severe cases of lung injuries, while children infected with SARS-CoV-2 frequently present milder forms of the disease [5,8].

COVID-19 disease has many clinical manifestations. For example, in adults, it varies from an asymptomatic disease to mild symptoms, not usually correlated with a respiratory illness [9], to severe infections with a high admission rate in ICU units, mainly due to comorbidities that potentiate the lethality of the infection [10]. The manifestations of SARS-CoV-2 infection in neonates and children can be asymptomatic [11] or can include mainly fever, lack of appetite, respiratory symptoms (such as cough and pharyngitis), diarrhea and lethargy [6,7]. 

Studies show that the laboratory findings are non-specific, but most of them include high levels of C-reactive protein, lymphopenia or normal white blood cell (leukopenia and leukocytosis may occur but are less common), high levels of MB-creatin kinase, increased D-dimer levels and abnormal hepatic probes (increased levels of AST, ALT, LDH) [6,7,8,12,13].

The primary changes that can be found using LUS in evaluating children with SARS-CoV-2 include:

- Transverse physiologic A-lines that depict healthy parenchyma;
- Isolated/sparse vertical B-lines are equivalent to interstitial edema;
- Confluent vertical B-lines correspond to alveolar edema;
- Subpleural/peripheral consolidations;
- Irregularities and thickening of the pleura [14,15,16].

A bilateral pattern of B-lines in association with subpleural consolidation and irregular pleural lines are the most characteristic ultrasonographic changes and correlate well with the findings of the pulmonary CT scan [17].

The lung ultrasound is an important tool both for the initial diagnosis of SARS-CoV-2 infection and for monitoring its evolution during the state period and after the patient’s healing. Thus, it has been demonstrated that in children with mild forms of SARS-CoV-2 infection, a higher ultrasound score persists than in children who have not had this infection, unlike children who have had an asymptomatic form, have the same ultrasound score as the children who have not contracted SARS-CoV-2 infection [15].

Some studies report correlations between the tomographic image of the lung in SARS-CoV-2 infection and the elevated alanine aminotransferase, D-dimer and leukocyte values [18].

This paper aims to find the main respiratory changes detected in SARS-CoV-2-infected infants and children under two years of age using the lung ultrasound technique. The study also aims to determine a correlation between lung injuries, clinical manifestations and laboratory findings in the infected infants.

## 2. Patients and Methods

The study to be further introduced was performed at The Clinic of Infectious Diseases II and the intensive care unit of the clinic, with beds available for the pediatric age groups, at ‘Dr. Victor Babes’ Clinical Hospital of Infectious Diseases and Pneumophthisiology in Timisoara between November 2021 and the end of October 2022. This study is an analytical one of a retrospective cohort, with consecutive sampling.

The inclusion criteria used in the selection of patients were the following:

- Infants with SARS-CoV-2 infection that were hospitalized for more than two days;
- Children under 2 years of age with SARS-CoV-2 infection who had been hospitalized for more than two days.

The exclusion criteria were the following:

- Infants and children with SARS-CoV-2 infection that were hospitalized for less than two days;
- Infants and children with chronic lung diseases such as bronchopulmonary dysplasia, cystic fibrosis, immunodeficiency, etc.;
- Children over the age of 2 years with SARS-CoV-2 infection;
- Infants and children lacking informed consent from parents or legal guardians.

The children included in the study were diagnosed with SARS-CoV-2 infection according to their Polymerase Chain Reaction (PCR) result test. All the subjects were tested using a Real-Time Multiplex PCR Test, which includes Adenovirus, Coronavirus 229E, HKU1, NL63, OC43, Human metapneumovirus, Human rhinovirus/enterovirus, Human respirovirus 1, 2, 3 and 4, Respiratory Syncytial Virus, Bordetella parapertussis, Bordetella pertussis, Chlamydia pneumoniae, Mycoplasma pneumoniae, Influenza A and B, MERS-CoV and SARS-CoV-2. The children with other co-infections were ruled out.

The decision to hospitalize the patients was based on the clinical symptoms presented by the patients (psychomotor agitation, asthenic syndrome, fever, cough, rhinorrhea and acute dehydration syndrome). Three children tested positive for PCR in an emergency care unit at a pediatric hospital and, depending on the symptoms, were redirected to this health unit.

**Figure 1:**
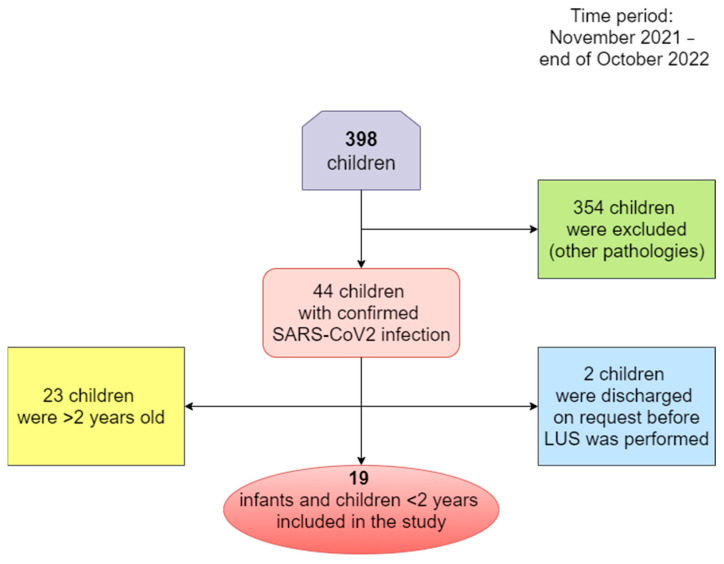
The algorithm of subjects’ selection and exclusion criteria.

Children under two years are not able to express symptoms, and objectification is sometimes difficult; therefore, complications might rapidly occur in this age group. Thus, at this vulnerable age, it was decided to closely follow the evolution of these cases according to the hospital protocols. This is, therefore, the rationale behind the decision to follow the ultrasound changes of children under the age of 2. The criteria listed above are presented in the following scheme—Figure 1.

The analyzed data was extracted from the hospital’s computer program—virtual archive (InfoWorld), and stored in a Microsoft Office Excel table, which helps to perform a better comparison and organization of patient records. The most significant parameters that were considered for the analysis were:

- Gender;
- Age (months);
- Anthropometric measurements (weight of children);
- Number of positive PCR tests;
- Days of hospitalization/days of convalescence;
- Signs and symptoms of the infection (psychomotor agitation, asthenic syndrome, fever, cough, rhinorrhea, acute dehydration syndrome, vomiting, diarrhea, nasal obstruction, dysphagia, dysphonia, loss of appetite, dyspnea, oropharyngeal candidiasis, presence of inflammatory lymph nodes, presence of congestive pharynx);
- Other associated pathologies;
- Biological markers and inflammatory probes (hemoglobin, leukocytes, lymphocytes, neutrophils, monocytes, thrombocytes, erythrocyte sedimentation rate, procalcitonin, C-reactive protein, ferritin, LDH, hepatic transaminases, bilirubin, D-dimer level, fibrinogen, interleukin-6);
- Bacterial and fungal cultures;
- Imaging examinations;
- Score of lung affection based on ultrasound.

We chose to divide the subjects into two groups—those that presented respiratory disease and those without respiratory complications because most infants and children had mild types of the disease and did not present serious additional respiratory signs and symptoms. Because patients with other respiratory diseases and infections were ruled out of the study, the selected lot included only infants and children with criteria of pneumonia.

In all the cases, the lung ultrasound examination was performed in the initial days (from the 2nd to the 4th day) of the patient’s hospitalization by a radiologist with a minimum of three years of experience in lung ultrasound in newborns, children and adults, validated by a senior radiologist with nine years of experience. The examinations were conducted using a portable General Electric Vivid IQ with linear probe 9L-RS [2.4–10.0 MHz] and convex probe 4C-RS [1.5–5.0 MHz], and an ultrasound system Philips EPIQ 5 with L12-5 50 mm linear array [12-5 MHz]. 

A 12-area score was accounted for each infant or child admitted to the hospital. This score is similar to the one described by Mongodi et al. and used for COVID-19-associated pneumonia in neonates—lung ultrasound score (LUSS) [6,19]. There were 6 areas on each hemithorax (2 anterior, 2 lateral and 2 posterior) divided by the nipple line. Each area explored was scored from 0 to 3 points, depending on the aspect of artifacts and the presence or absence of subpleural consolidation. The LUSS was summarized in the table below—Table 1 [6,19].

All data and analyses were processed with a licensed version of MedCalc^®^ Statistical Software version 20.026 (MedCalc Software Ltd., Ostend, Belgium; https://www.medcalc.org; (accessed on 22 December 2022)).

The plot distribution was analyzed by applying the Shapiro–Wilk test. According to these results, the following parameters—age (months), weight, days of hospitalization and convalescence, thrombocytes, LDH, urea, fibrinogen and LUSS stayed within normal values—parametric distribution. For the rest of the parameters, the Shapiro–Wilk test showed a significant departure from normality, which required the application of non-parametric tests.

The central tendency indicators were arithmetic mean and dispersion—standard deviation (S.D.) for parametric variables and medians and interquartile range [IQR] for the non-parametric ones. The relationship between the symptoms and the lung ultrasound score was documented through statistical tests, the Mann–Whitney U test and cross tabs for better illustration. The difference between the medians was demonstrated using the Mann–Whitney U test. Moreover, Spearman’s rank correlation rho between LUSS, biomarkers of inflammation and symptoms was calculated based on the degree of correlation: with rho near ±1, the correlation is perfect; very strong—rho between ±0.80 to 1; strong correlation with rho between ±0.60 to ±0.79; moderate one with rho between ±0.40 and ±0.59; weak correlation with rho between ±0.20 and ±0.39; and very weak correlation with rho under ±0.19. The *p*-value *p* < 0.05 was considered significant.

The study was conducted in accordance with the Declaration of Helsinki and approved by the ethics committee of ‘Dr. Victor Babes’ Clinical Hospital of Infectious Diseases and Pneumophthsiology in Timisoara (number 10289/25 October 2021).

## 3. Results

### 3.1. Demographic Data

Out of a total of 19 infants and children with SARS-CoV-2 infection who were admitted at The Clinic of Infectious Diseases II and the intensive care unit of the clinic at ‘Dr. Victor Babes’ Clinical Hospital of Infectious Diseases and Pneumophthisiology in Timisoara, 10 infants and children are of female gender (52.63%). Twelve of them (63.15%) are infants, while 36.84% are children under the age of two years (24 months). 

The mean age is 11.21 ± 7.70 months (presented as mean ± standard deviation—S.D), with a minimum of 1 month and a maximum of 24 months, with the median age being 10 months.

All demographic data are summarized in the table below—Table 2.

### 3.2. Clinical and Biological Markers of COVID-19 Infection in Infants and Children under 2 Years

Table 3 presents the most relevant signs and symptoms analyzed for infants and children, while Table 4 shows biomarkers and paraclinical data for those with SARS-CoV-2 infection, presented as the mean, standard deviation or median and [IQR].

### 3.3. Lung ultrasound investigation, score and correlation

In all cases of SARS-CoV-2 infection, the lung ultrasound technique was used to examine lung injuries. This method was chosen in accordance with the ALARA principles, meaning that no other imaging or radiological methods were employed. Additionally, the infant and children’s overall health did not necessitate any additional imaging investigation. The lung ultrasound examination was performed in the first days (from the 2nd to the 4th day) of hospitalization. 

**Figure 2:**
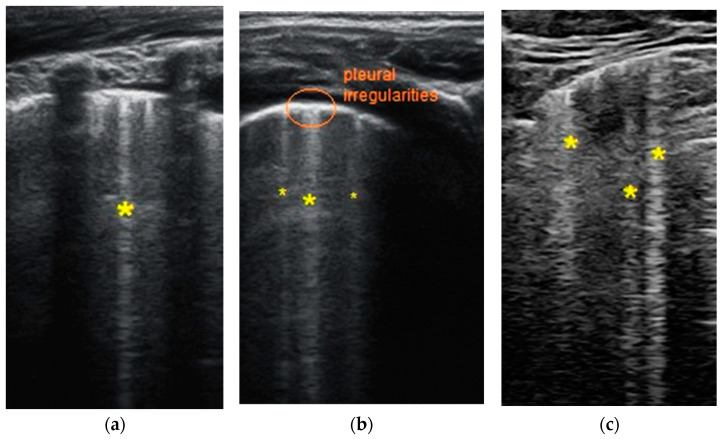
The lung ultrasound showed (**a**,**c**) sparse B-lines (yellow asterisk *) with small zones of pleural irregularities corresponding to a LUSS = 1 and (**b**) sparse B-lines (yellow asterisk) with small zones of pleural irregularities corresponding to a LUSS = 1.

**Figure 3:**
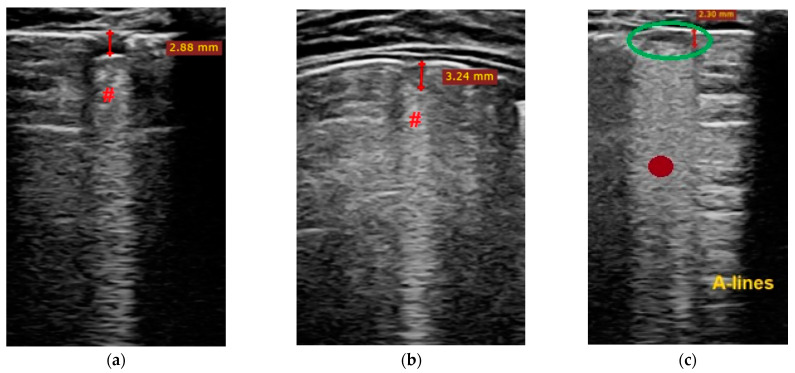

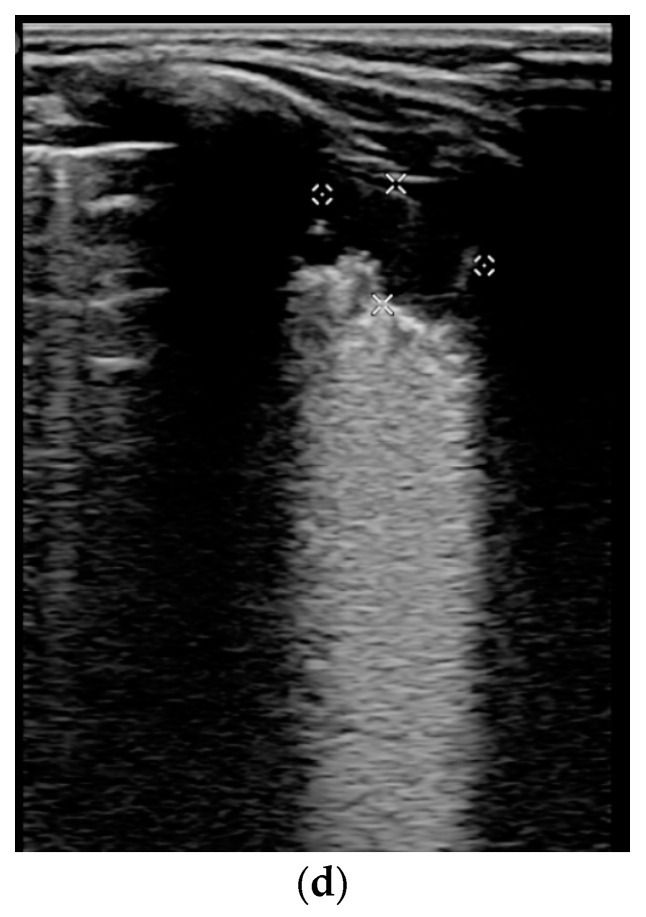
(**a**–**c**) The lung ultrasound showed a small consolidation area (red hash #) with a length < 1 cm and with associated pleural abnormalities and confluent B-lines corresponding to a LUSS = 2. (**c**) Illustrates a small area of consolidation (area within the green circle) associated with confluent B-lines (burgundy filled circle), LUSS = 2. (**d**) The lung ultrasound shows a large consolidation area with dimensions of 1.5/1.03 cm associated with pleural abnormalities and confluent B-lines corresponding to a LUSS = 3. This image is from an infant with bacterial pneumonia, not included in this study.

Each patient’s LUSS (LUS score) ranges from 1–8 points, with a maximum score of 36 points. The average score is 4.47 ± 2.36 (S.D.), and the median score is 4, with a range of 2.25 to 6.75. The region with the highest score is the posterior right inferior area (R6), with three patients receiving a score of 2 and thirteen patients receiving a score of 1, totaling 19 points. The second-highest score was in the posterior left inferior area (L6), with one patient receiving a score of 2 and three patients receiving a score of 1, totaling 5 points. The lowest scores were recorded in the anterior left areas. The main changes described by thoracic ultrasound that appeared in a minimum of one area/children or infants were:

- Sparse B-lines (Figure 2a,b)—100% (*n* = 19);
- Confluent or coalescent B-lines (Figure 3a)—36.84% (*n* = 7);
- Pleural abnormalities (irregularities, thickening, fragmented)—42.10% (*n* = 8);
- Subpleural consolidation < 1 cm (Figure 3b)—21.05% (*n* = 4);
- Pleural effusion—5.26% (*n* = 1);
- Without large consolidation.

**Figure 4:**
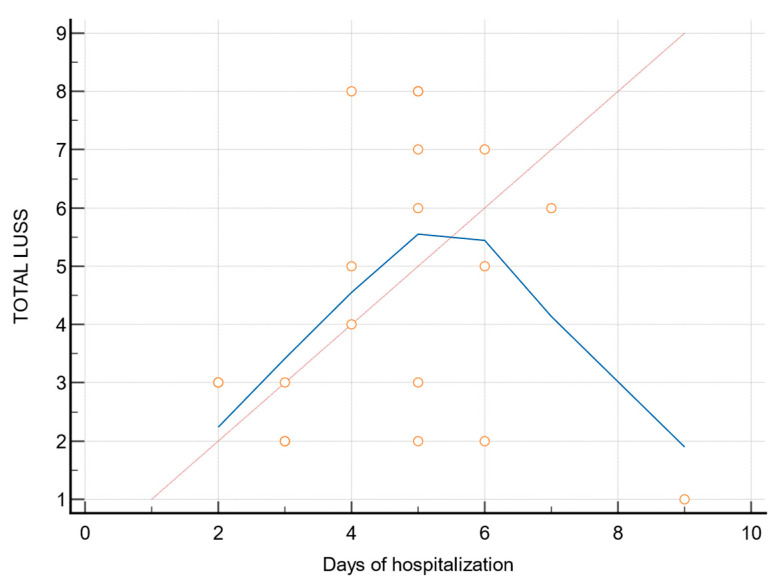
The relationship between days of hospitalization and LUSS. Red dots were used to indicate the correspondence days of hospitalization and LUSS. The blue trendline was used to show the pattern or trend more clearly and to demonstrate that there was no association found between days of hospitalization and the LUSS score. The red trendline illustrates an ideal positive linear correlation.

The figure below (Figure 4) shows there is no association between days of hospitalization and LUSS score rho = 0.14 and *p* = 0.54. In conclusion, there was no association found between days of hospitalization and the LUSS score. 

The test applied is the Mann–Whitney U test (independent samples)—for the calculation of *p*-value for days of convalescence, and LUSS (creating the two groups mentioned above) shows no statistical difference, meaning that there is no change in days of convalescence by the LUSS score *p* = 0.59, though there is no correlation between those two (LUSS and days of convalescence) rho = 0.008 and *p* = 0.97.

In the table below, the LUSS value is divided into groups according to the Mann–Whitney U test—Table 5.

**Figure 5:**
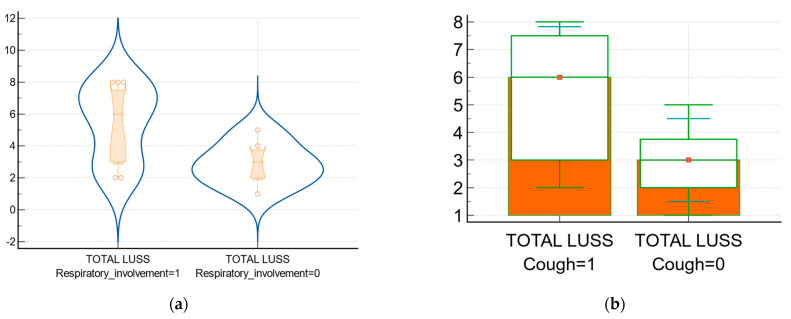
The relationship between respiratory involvement and LUSS: 1 means presence/affirmative, and 0 means absence/negative. (**a**) The frequencies chart of children with/without respiratory involvement in correlation with the LUSS score (notched box-and-whisker, violin representation with dots that plot all data); (**b**) Box-and-whisker of data comparison between LUSS at infants and children with cough and LUSS of infants and children without cough.

The relationship between respiratory involvement and LUSS is represented graphically in Figure 5a. Also, the relationship between cough and LUSS is represented in Figure 5b.

Spearman’s rank correlation rho or Pearson correlation coefficient r between the LUSS and the main biomarkers of inflammations/infections are presented in Table 6.

## 4. Discussion

Fortunately, in this study, none of the children included in the research required orotracheal intubation or oxygen administration due to the fact that all of them had mild to moderate forms of the disease. There are few studies like the one conducted by Jackson et al., which concluded that chances of developing severe illness and mortality rates are low in children [20]. Moreover, one multicenter study that included a number of 91 children cases with COVID-19-associated pneumonia concluded that it is unnecessary to perform invasive or irradiating chest imaging in children because most of their evolution will be with mild symptoms, mostly fever and cough [8]. This fact was the motivation of our study for investigating infants and small children with a non-invasive and non-irradiating method, such as the lung ultrasound technique. However, it should be noted that the thoracic ultrasound is limited to exploring only the subpleural area, with a satisfactory resolution within the first four centimeters below the pleural line [21]. Many studies have concluded that the peripheral spaces were the most affected by COVID-19-associated pneumonia, both in children and adults, so LUS can be suitable for investigating these areas [4,5,8,14,22].

Unlike adults, who have a higher risk of developing aggressive forms of the disease and require more hospitalization days, most of the children in our study needed less than 5 days of hospital admission [23]. Furthermore, the short hospital stay was also because more than half of the mothers or caregivers decided to be discharged against medical advice (DAMA). The test applied for the calculation of *p*-value for days of hospitalization and LUSS (creating two groups for LUSS, one with a score < 4 and one group with a score > 3) showed no statistical difference, meaning that there is no change in days of hospitalization based on the LUSS score. Additionally, there is no correlation between those two (LUSS and days of hospitalization). The most common symptoms our patients developed were fever, mild acute dehydration syndrome, congestive pharynx, cough, loss of appetite and altered general condition (moderate to slightly severe). Similar results were obtained by Mansourian et al. in a review that included 32 articles published on this topic [24]. On the other hand, diarrhea with bloody stools and dyspnea were present in only one patient from our study, while runny nose and red eyes were found in 10.52% of the patients. These results are similar to the ones found by Mansourian and colleagues [24]. Moreover, a large study involving more than 5000 children and more than 1000 infants and children under 2 years old revealed a prevalence of fever in the age group < 1 year and 1–4 years from 64.8–77.2%, which is comparable with our results [25]. Additionally, cough was found with a prevalence rate of 53.1–65.5% in their study, which is similar to our findings [25]. Regarding pulmonary involvement, the infants and children of older age had an alteration in their respiratory status compared with early age.

The clinical parameters and symptoms for defying pulmonary involvement, like cough, had a similar imaging value for lung lesions, according to the Mann–Whitney U test result. Thus, the children with such symptoms (respiratory involvement) had a median of LUSS higher than the median of LUSS in the ones without symptoms (3), which is statistically significant.

The inflammatory probes revealed the biological status of infants and children. They were analyzed for all intents and purposes, taking into account the leukocytes, lymphocytes, neutrophils, monocytes, thrombocytes, ESR, LDH, AST, CRP, fibrinogen, procalcitonin, ferritin, D-dimer and IL-6. From all laboratory findings, LDH, D-dimer and IL-6 levels were correlated with the lung ultrasound score with higher statistical evidence [26,27]. Additionally, higher IL-6 levels were found in patients with less severe forms of SARS-CoV-2 infection, according to Salton and colleagues [26]. The children included in our study developed a mild to moderate form of SARS-CoV-2 infection, which is similar to the results of the study conducted by Salton et al. regarding the higher IL-6 level in this grade of severity. Unfortunately, we did not have the possibility to evaluate other cytokines levels (IL-8, IL-9, G-CSF, IP-10, MCP-1, etc.), which were proved to be higher in severe cases [26].

The strong positive correlation between the LUSS and LDH levels for symptomatic infants and children (respiratory involvement) demonstrates that inflammatory markers, along with the ultrasound score, can outline the patient’s prognosis and evolution. Additionally, the strong positive correlation between the LUSS and D-dimer levels for all subjects included and between LUSS and D-dimer levels for symptomatic ones (respiratory involvement) complements these ideas. Additionally, the same correlation was demonstrated between the LUSS and IL-6 levels, a fact already proved in the study based on newborns with COVID-19-associated pneumonia. Despite the small sample, D-dimer levels were correlated with LUSS in symptomatic patients, while similar results were also found in the entire lot. In addition, D-dimer levels were significantly correlated with the severity of SARS-CoV-2 infection [13]. Even if there is a correlation, because of the low data, there may be high BIAS as the concordance correlation coefficient (pc) is low [6].

Regarding the blood parameters, they were compared with another study that included children. The mean value of leukocytes for our study was higher compared with 8880 ± 1086 in a study conducted by Musolino et al. The mean value of neutrophils in the present study is lower compared with a higher mean value of 7023 ± 996. In addition, the lymphocyte mean value is higher compared with a considerably lower mean value of 1057 ± 112. Regarding the inflammatory status, CRP levels are lower (11.44 ± 1.8) compared with the study’s result of 20.56 ± 46.22 mg/L (high standard deviation because of one outlier value of 191 mg/L). These variations could be caused by a multisystemic inflammatory disease during SARS-CoV-2 infection and other associated pathologies of the children included [22]. The blood levels of neutrophils, AST and IL-6 were higher in the group of patients with pulmonary involvement but with weak evidence due to the small sample. In addition, these biomarkers suggest the inflammatory status of the patients, which was correlated with LUSS. Moreover, an increased IL-6 level was reported by Manti et al. in their review of the SARS-CoV-2 infection in the pediatric population [13]. Another interesting fact was the higher level of total bilirubin in the group of infants with respiratory involvement, with no other relevant evidence found in the literature.

The relationship between LUSS and O_2_ saturation was a strong linear negative one, which can be considered an important parameter that must be explored in respiratory pathologies, especially in COVID-19-associated pneumonia in infants and small children. Moreover, a similar strong negative correlation was found in the study conducted on newborns with SARS-CoV-2 infection [6]. 

According to Buonsenso and Vetrugno, lung ultrasound is becoming a reliable imaging method in respiratory pathologies for both adults and children [5]. Moreover, Vetrugno et al. described four different types of evolution, comparing the symptoms of patients and their imaging progress [28]. One of these phenotypes, specifically the 1st and 4th ones, seems to be consistent with what happens to infants and small children. The 1st phenotype describes subjects whose clinical improvement is independent of the LUS progression. In contrast, the 4th phenotype includes patients with an improvement in their clinical condition but without an obvious improvement in LUS of the injured lung and sometimes with an apparent aggravation. This may be the reason for a strong correlation between the LUSS and just a few of the inflammatory biomarkers demonstrated in this study (LDH, D-dimer, IL-6) [28].

The appearance of sparse B-lines in a minimum of one evaluated area was the most common finding in the lung ultrasound performed on the selected subjects, with a prevalence of 100%. These results are similar to the results obtained by the study carried out by Musolino et al. and the study that included newborns with COVID-19-associated pneumonia, with the same prevalence of sparse B-lines [4,6,22]. Another systematic review found a prevalence of sparse B-lines of 50% despite the subjects’ condition (6.81% asymptomatic and 81.81% with mild to moderate symptoms) [14]. 

The aspect of confluent or coalescent B-lines is lower than the prevalence obtained in the study by Musolino et al. (80%), defined as multiple/severe B-lines, and also lower than the category described as white lung (50%) [22]. Additionally, this discrepancy in the results could be because of the severity of the included subjects with an additional difference between the LUSS mean value of 10.5 ± 1.81 compared to our study (4.47 ± 2.36). However, our result is in accordance with Caroselli et al., who found in their review a prevalence of coalescent B-lines of 25% [14]. Additionally, pleural abnormalities (irregularities, thickening, fragmented) are pretty much similar to Caroselli et al.’ review (42.10% in our study compared to 34.09% for pleural irregularities +4.55% described as thickening of the pleural line in their review) [14].

Many studies found that pleural effusion in the evolution of COVID-19-associated pneumonia in children is a very rare alteration that appeared in the lung ultrasound technique [6,8,14]. Our study strengthens these results. 

Many studies have concluded that the peripheral spaces from posterior inferior areas are most affected by COVID-19-associated pneumonia, both in children and adults [4,5,8,13,14,22]. The mean value of LUSS in our study was higher in the posterior right inferior area and the posterior left inferior area, in accordance with the results presented above. This imaging aspect has become a defining one for the infection of SARS-CoV-2 and COVID-19 pneumonia [4,5,8,14,22].

Furthermore, in comparison with the results presented by Manti et al., the variations of inflammatory parameters are pretty much similar, with increased serum levels of IL-6, LDH and D-dimers in enrolled subjects. An increased procalcitonin level was associated with a bacterial superinfection in infants and children with SARS-CoV-2 infection. Additionally, they concluded that the peripheral and subpleural spaces of the lower lobes are the most affected areas, results that are similar to the ones already presented [13].

Another situation in which LUS is the preferred imaging method in evaluating COVID-19 patients is pregnant women, in whom exposure to X-rays or CT scans could have a negative impact on the fetus, so it should be avoided [29]. A study by Vetrugno et al. shows that LUS is an adequate method for monitoring pregnant women with SARS-CoV-2 infection, having a good correlation with respiratory symptoms [30]. 

An important fact was proved by Ruaro et al. in their study regarding the correlation between LUS and HRCT (high-resolution computed tomography) in patients with IDL (interstitial lung disease) [31]. This is significant in monitoring children with ‘long-COVID’ syndrome since studies have indicated the presence of persistent, fibrotic-like alterations in the lung parenchyma of individuals with SARS-CoV-2 infection over time [32,33].

All in all, the low sample size is an important factor in the interpretation of study results. Even if the correlation between LUSS and D-dimer level, LUSS and O_2_ saturation was strong, and there is some proof of an impact, the outcome did not manage to reach statistical significance. Instead, larger confirmatory studies should be developed using the findings from the present research.

### 4.1. Limitation of Study/Weakness

One of the limitations of the study is the number of subjects included in the research. For this reason, the results of the study should be interpreted within the present limitations—despite some evidence of an impact, the results did not provide a high significance level. A more significant statistical result would be found if the group of patients was larger, and the correlations would have been better and stronger.

Another limitation is the fact that while most of the patients did not come back for follow-up, some of them were even discharged earlier on request, contradicting the treating physician’s advice. The main reason was that, with mild forms of the condition, the evolution of the patients was favorable, and parents/relatives did not understand the importance of additional check-ups. Monitoring through ultrasound the changes found on the admission would be beneficial. 

### 4.2. Further Directions 

Several future directions can be implemented, such as larger, multicentric studies, in order to find stronger correlations between lung ultrasound changes and biomarkers in SARS-CoV-2 respiratory disease. Another future direction that can be developed is the follow-up of patients with respiratory pathologies to observe the lung changes in dynamic, taking into account the fact that lung ultrasound is a non-irradiating imaging method and a repeatable one. Moreover, according to Buonsenso and Vetrugno, important steps have already been taken to make LUS a reliable tool in lung changes associated with respiratory pathologies [5]. The next step is to find a score of severity based on LUSS, biomarkers and clinical symptoms for a better evaluation of the patients.

## 5. Conclusions

Infants and children under the age of two are more likely to develop mild forms of COVID-19 disease, even if inflammatory markers, such as LDH, D-dimer and IL-6, have elevated blood levels. The blood levels of neutrophils, AST and IL-6 were higher in the group of patients with pulmonary involvement.

The predominant lung abnormalities observed by LUS were sparse B-lines and confluent or coalescent B-lines, implying that this imaging method can be utilized to investigate infants and children with mild manifestations of the condition. The infants and children with respiratory involvement had a higher LUSS. Nevertheless, there is no change in days of convalescence and hospitalization by the LUSS. 

Despite the small sample, D-dimer levels were correlated with LUSS in patients with respiratory involvement, while similar results were also found in the entire lot. Furthermore, the relationship between LUSS and O_2_ saturation was a moderate-strong linear negative one.

## Figures and Tables

**Figure 6:**
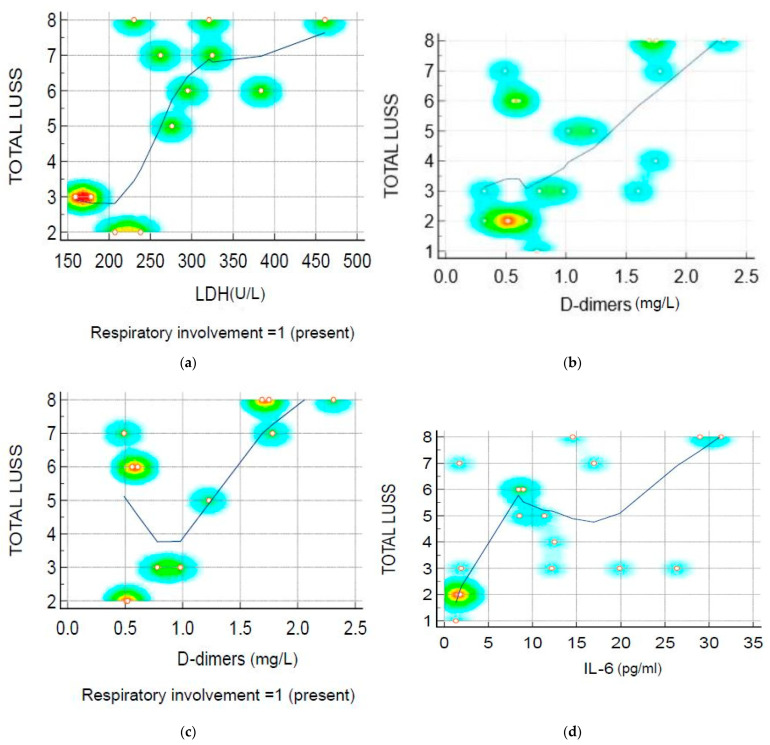
Scatter diagram with heat map of correlation between LUSS and biomarkers: (**a**) LUSS and LDH levels of infants and children who presented respiratory involvement; (**b**) LUSS and D-dimer levels from all subjects; (**c**) LUSS and D-dimer levels from the subjects who presented respiratory involvement; (**d**) LUSS and IL-6 levels—all positive linear correlation. The heat map was used to indicate the density of points, suggesting clusters of observation (red means the most interaction, while blue means the least). The LOESS (Local Regression Smoothing) trendline was used to show the pattern or trend more clearly and to demonstrate the positive linear correlation.

**Figure 7:**
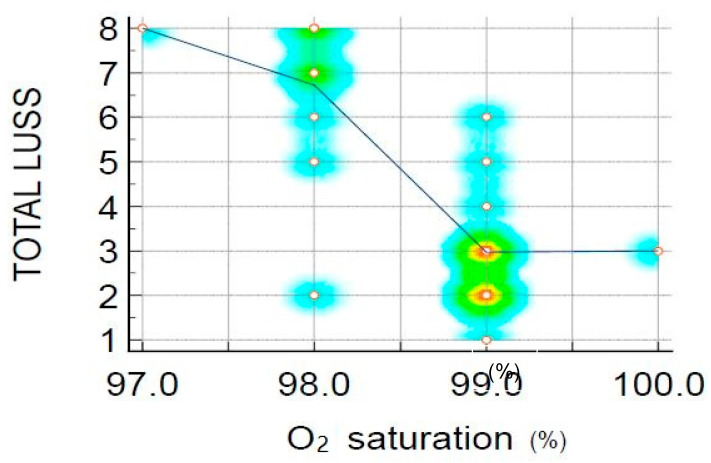
Scatter diagram with heat map of correlation between LUSS and O_2_ saturation—negative linear correlation. The heat map was used to indicate the density of points, suggesting clusters of observation (red means the most interaction, while blue means the least). The LOESS (Local Regression Smoothing) trendline was used to show the pattern or trend more clearly and to demonstrate the negative linear correlation.

**Table 1:** LUSS for each area of interest examined.

LUSS = 0 Points	LUSS = 1 Point	LUSS = 2 Points	LUSS = 3 Points
Normal/physiological A-lines	More than 2 B-lines (sparse B-lines) with associated pleural abnormalities	Coalescent or confluent B-lines	Large peripheral consolidation (wider than 1 cm) in association or not with air bronchogram
One or two B-lines per intercostal space		‘White-lung’ aspect or small peripheral consolidation (smaller than 1 cm)	

**Table 2:** The baseline characteristics of infected infants and children presented as mean ± S.D., Min, Max values or percentages.

Infants and Children’s Characteristics	Entire Lot *n* = 19	Patients with Respiratory Involvement (*n* = 12)	Patients without Respiratory Involvement (*n* = 7)	Independent Samples Tests
Age (Months) Mean ± S.D. Min; Max	11.21 ± 7.70 1; 24	13.16 ± 8.33 2; 24	7.87 ± 5.49 1; 18	Test Statistic t = −1.49 *p* = 0.15
Weight (Kilograms) Mean ± S.D. Min; Max	9.04 ± 3.08 3.5; 13.6	9.63 ± 2.95 4.5; 13.5	8.02 ± 3.26 3.5; 13.6	Test Statistic t = −1.09 *p* = 0.28
Total PCR Tests Per Child Median; [IQR] Min; Max	1; [1;2] 1; 3	1.5; [1;2] 1; 3	1; [1;2] 1; 3	Mann–Whitney U = 39 *p* = 0.77
Days of Hospitalization Per Child Mean ± S.D. Min; Max	4.68 ± 1.73 2; 9	5 ± 1.20 3; 7	4.14 ± 2.41 2; 9	Test Statistic t = −1.04 *p* = 0.31
Days of convalescence per child Mean ± S.D. Min; Max	7.68 ± 2.35 4; 13	7.75 ± 2.37 5; 13	7.57 ± 2.50 4; 12	Test Statistic t = −1.55 *p* = 0.87
Second Positive PCR tests	57.9% (*n* = 11)			

**Table 3:** The symptoms and comorbidities of infants and children with SARS-CoV-2 infection presented as the number of patients and percentage (%).

Signs and Symptoms in Infants and Children	*n* = 19 (Percentage %)	Patients with Respiratory Involvement *n* = 12 (Percentage % of the Entire Lot)	Patients without Respiratory Involvement *n* = 7 (Percentage % of the Entire Lot)
Moderately influenced general condition	11 (57.89)	8 (42.10)	3 (15.78)
Slightly influenced general condition	8 (42.10)	4 (21.05)	4 (21.05)
Psychomotor agitation	3 (15.78)	2 (10.52)	1 (5.26)
Asthenic syndrome	8 (42.10)	6 (31.57)	2 (10.52)
Fever (≥37.5 °C)	16 (84.21)	10 (52.63)	6 (31.57)
Rhinorrhea	7 (36.84)	3 (15.78)	4 (21.05)
Nasal obstruction	8 (42.10)	5 (26.31)	3 (15.78)
Congestive pharynx	14 (73.68)	8 (42.10)	6 (31.57)
Dysphonia	4 (21.05)	2 (10.52)	2 (10.52)
Dysphagia	2 (10.52)	2 (10.52)	0
Red eyes and runny nose	2 (10.52)	2 (10.52)	0
Mild acute dehydration syndrome (<5% of weight)	15 (78.94)	9 (47.36)	6 (31.57)
Episodes of diarrhea	3 (15.78)	3 (15.78)	0
Diarrhea with bloody stools	1 (5.26)	1 (5.26)	0
Vomiting	4 (21.05)	3 (15.78)	1 (5.26)
Loss of appetite	11 (57.84)	8 (42.10)	3 (15.78)
Lateral-cervical lymph nodes	3 (15.78)	2 (10.52)	1 (5.26)
Dyspnea	1 (5.26)	1 (5.26)	0
Glottis spasm	1 (5.26)	1 (5.26)	0
**Associated pathologies and comorbidities**			
Oral candidiasis	3 (15.78)	2 (10.52)	1 (5.26)
Lactose intolerance	2 (10.52)	2 (10.52)	0
Egg intolerance	1 (5.26)	1 (5.26)	0
Seizures	1 (5.26)	1 (5.26)	0
Repeated otitis	1 (5.26)	1 (5.26)	0
Atopic dermatitis	1 (5.26)	0	1 (5.26)
History of whooping cough	1 (5.26)	1 (5.26)	0
Urinary tract infection	1 (5.26)	0	1 (5.26)

**Table 4:** The biomarkers and paraclinical data of infants and children with SARS-CoV-2 infection presented as mean ± S.D. or median and [IQR], Min, Max values.

Biomarker (Unit Measurement)	Mean ± S.D. or Median; [IQR]	Patients with Respiratory Involvement (*n* = 12)	Patients without Respiratory Involvement (*n* = 7)	Independent Samples Tests
Hemoglobin (g/dL) Min; Max	11.50; [10.92; 12.10] 9.7; 12.7	11.50; [10.35; 12.15] 9.7; 12.7	11.50; [11.22; 12.05] 9.7; 12.5	Mann–Whitney U = 39 *p* = 0.79
Leukocytes (×10^9^/L) Min; Max	7890; [6400; 11062.50] 4230; 31,700	8695; [6170; 14760] 4230; 31,700	7380; [6697.50; 8100] 5440; 12,360	Mann–Whitney U = 34 *p* = 0.49
Lymphocytes (×10^9^/L) Min; Max	3960; [2427.5; 5787.5] 1060; 12,460	4140; [2180; 5720] 1060; 12,460	3120; [2815; 5655] 1800; 7760	Mann–Whitney U = 41 *p* = 0.93
Neutrophiles (×10^9^/L) Min; Max	2410; [1512.5; 4685] 720; 22,270	3755; [1700; 6010] 1220; 22,270	1710; [965; 3360] 720; 5050	Mann–Whitney U = 23 *p* = 0.10
Monocytes (×10^9^/L) Min; Max	1260; [797.5; 1697.5] 360; 3440	1635; [700; 2635] 360; 3440	1210; [967.5; 1402.5] 640; 1700	Mann–Whitney U = 33 *p* = 0.44
Thrombocytes (×10^9^/L) Min; Max	360,000.00 ± 124,869.26 138,000; 589,000	348,750.00 ± 106,279.32 222,000; 574,000	379,285.71 ± 159,316.99 138,000; 589,000	Test statistic t = 0.50 *p* = 0.62
Erythrocyte Sedimentation Rate (mm/h) Min; Max	10; [10; 15] 5; 70	12.50; [9; 17.50] 5; 70	10; [10; 15] 5; 60	Mann–Whitney U = 40 *p* = 0.86
LDH (U/L) Min; Max	279.10 ± 74.29 159; 461	278 ± 86.57 159; 461	281 ± 53.02 210; 361	Test statistic t = 0.08 *p* = 0.93
AST (U/L) Min; Max	53; [31.40; 65.35] 20.4; 160.1	60.40; [33.95; 81.15] 20.4; 160.1	34.70; [26.95; 57.87] 26.3; 60.6	Mann–Whitney U = 23 *p* = 0.10
CRP (mg/L) Min; Max	5.57 [2.63; 8] 0.4; 191	4.44 [2.65; 8.34] 0.4; 89.42	5.57 [2.96; 7.75] 0.66; 191	Mann–Whitney U = 41 *p* = 0.93
Fibrinogen (g/L) Min; Max	3.41; [2.90; 4.04] 1.8; 6.2	3.66; [3.37; 4.01] 1.8; 6.2	2.94; [2.78; 4.28] 2.67; 5.05	Mann–Whitney U = 29.50 *p* = 0.29
Procalcitonin (ng/mL) Min; Max	0.18; [0.08; 0.27] 0.05; 2.1	0.19; [0.09; 0.28] 0.07; 0.44	0.17; [0.09; 0.18] 0.05; 2.1	Mann–Whitney U = 35 *p* = 0.55
Ferritin (μg/L) Min; Max	91.68; [55.82; 147.17] 0.77; 1087	106.50; [58.73; 176.62] 0.77; 1087	91.68; [59.87; 122.37] 34.29; 524.87	Mann–Whitney U = 35 *p* = 0.55
D-dimer (mg/L) Min; Max	0.78; [0.53; 1.66] 0.32; 2.31	0.88; [0.54; 1.72] 0.49; 2.31	0.76; [0.40; 1.45] 0.32; 1.74	Mann–Whitney U = 34 *p* = 0.49
IL-6 (pg/mL) Min; Max	8.94; [ 1.73; 16.35] 1.32; 31.3	12.95; [ 5.08; 23.12] 1.40; 31.3	1.92; [ 1.55; 11.25] 1.32; 12.49	Mann–Whitney U = 22 *p* = 0.09
ALT (U/L) Min; Max	22.70; [14.27; 30.60] 9; 85	25.95; [13.50; 49.85] 9; 85	22.30; [16.47; 25.25] 13.10; 29.7	Mann–Whitney U = 34.50 *p* = 0.52
Urea (mg/dL) Min; Max	20.78 ± 6.36 12.3; 33.3	21.69 ± 6.70 13.4; 33.3	19.24 ± 5.89 12.3; 28.8	Test statistic t = −0.80 *p* = 0.43
Total Bilirubin (mg/dL) Min; Max	0.19; [0.11; 0.30] 0.07; 0.63	0.20; [0.15; 0.41] 0.11; 0.63	0.11; [0.09; 0.19] 0.07; 0.60	Mann–Whitney U = 21.50 *p* = 0.08

LDH = lactate dehydrogenase; AST = aspartate aminotransferase; ALT = alanine aminotransferase; CRP = C-reactive protein.

**Table 5:** LUSS value divided into groups according to the Mann–Whitney U test.

	Median LUSS, [IQR]	Average Rank LUSS	Mann–Whitney U Value	*p*-Value
Patients with respiratory involvement (*n* = 12) vs. patients without (*n* = 7)—Figure 5a	6 vs. 3 [3; 7.5] vs. [2;3.75]	12.12 vs. 6.37	16.50	0.02
Patients with fever (*n* = 16) vs. patients without fever (*n* = 3)	4.5 vs. 2	10.31 vs. 8.33	19	0.57

**Table 6:** Spearman’s rank correlation rho or Pearson correlation coefficient r between the LUSS and the main biomarkers of inflammations/infections.

Correlation between	Rank Correlation/Coefficient	95% CI	*p*-Value	Graphic Representation
LUSS and LDH at symptomatic patients (respiratory involvement)	rho = 0.60	0.04 to 0.87	0.03	Figure 6a
LUSS and D-dimer level	rho = 0.55	0.139 to 0.807	0.01	Figure 6b
LUSS and D-dimer level at symptomatic infants and children (with respiratory involvement present)	rho = 0.62	0.07 to 0.88	0.03	Figure 6c
LUSS and D-dimer level at symptomatic infants and children (with fever present)	rho = 0.50	0.01 to 0.80	0.04	
LUSS and IL-6 level	rho = 0.64	0.26 to 0.84	0.00	Figure 6d
LUSS and IL-6 level at symptomatic infants and children (with respiratory involvement present)	rho = 0.48	−0.11 to 0.83	0.10	
LUSS and IL-6 level at symptomatic infants and children (with fever present)	r = 0.45	−0.05 to 0.77	0.07	
LUSS and O_2_ saturation level	R = −0.65	−0.865 to −0.28	0.00	Figure 7

## Data Availability

Data available on request.
